# Supplementary material for: Signatures of Mitochondrial Dysfunction and Impaired Fatty Acid Metabolism in Plasma of Patients with Post-Acute Sequelae of COVID-19 (PASC)
Source: Metabolites. 2022 Oct 26;12(11):1026. doi: 10.3390/metabo12111026 (PMC9699059; doi:10.3390/metabo12111026)
Supplement: Supplementary file 1 [file metabolites-12-01026-s001.zip › metabolites-1993365-supplementary.pdf]

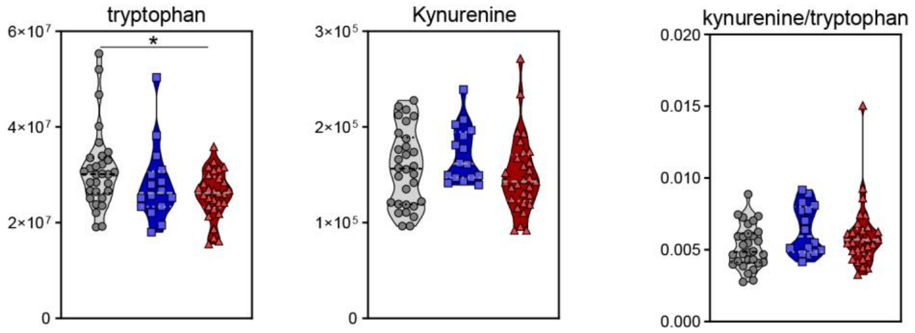

Figure S1. Tryptophan and kynurenine metabolism; * $p < 0.05$.

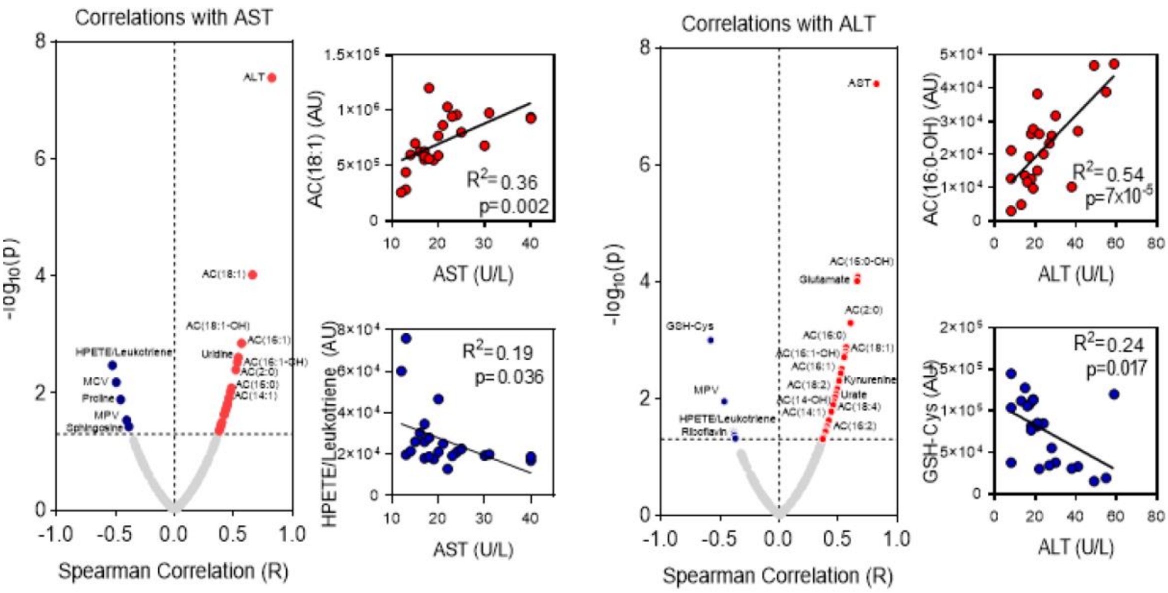

Figure S2: Clinical parameters and metabolites of PASC.
